# Pegylated Interferon Combined With Low-Dose Total Skin Electron Beam Therapy for Advanced Stage Mycosis Fungoides: Two Case Reports and Literature Review

**DOI:** 10.1016/j.adro.2024.101663

**Published:** 2024-10-28

**Authors:** Khaled Elsayad, Rudolf Stadler, Hans Theodor Eich

**Affiliations:** a Department of Radiation Oncology, University Hospital of Muenster, Muenster, Germany; b Department of Radiation Oncology, UKGM Marburg, Marburg, Germany; c Department of Radiation Oncology, Marburg Ion-Beam Therapy Center (MIT), UKGM Marburg, Marburg, Germany; d University Cancer Center (UCT) Frankfurt-Marburg, Marburg, Frankfurt, Germany; e Department of Dermatology, Johannes Wesling Medical Centre, University of Bochum, Minden, Germany

## Introduction

Radiation therapy (RT) is an effective option in mycosis fungoides (MF).^1^ Low-dose radiation regimens have demonstrated adequate disease control with a reasonable toxicity profile.^1^^,^^2^ The treatment approach for patients with early-stage MF is associated with long-term remission. However, advanced-stage MF is generally palliative except on some rare occasions following bone marrow transplantation.^1^, ^2^, ^3^, ^4^ Nevertheless, the responses in advanced stages are usually brief.^3^, ^4^, ^5^ Recent reports suggest that low-dose total skin electron beam therapy (TSEBT) combined with maintenance treatments may improve clinical outcomes and prolong the response durations.^6^, ^7^, ^8^, ^9^

Interferons (IFNs) are natural polypeptides orchestrated by eukaryotic cells after stimulation by various factors and exert cytotoxic and immunomodulatory effects.^10^ The common types of IFNs are produced by recombinant DNA technology.^10^ Although randomized trials evaluating the use of IFNs in MF are lacking, investigators have demonstrated their effectiveness in different clinical scenarios and disease stages.^11^, ^12^, ^13^ Regarding the mechanism of action, IFN alpha ameliorates several immune defects and activates CD8+ T cells and natural killer cells. Additionally, IFNs can augment cytotoxicity by releasing danger signals.^11^ Conceptually, the consideration of combination therapy of advanced-stage MF patients is reasonable. This publication reports the effectiveness and tolerability of low-dose TSEBT and pegylated IFN alpha-2a combination therapy in 2 cases with advanced-stage MF.

## Case Report

**Figure 1:**
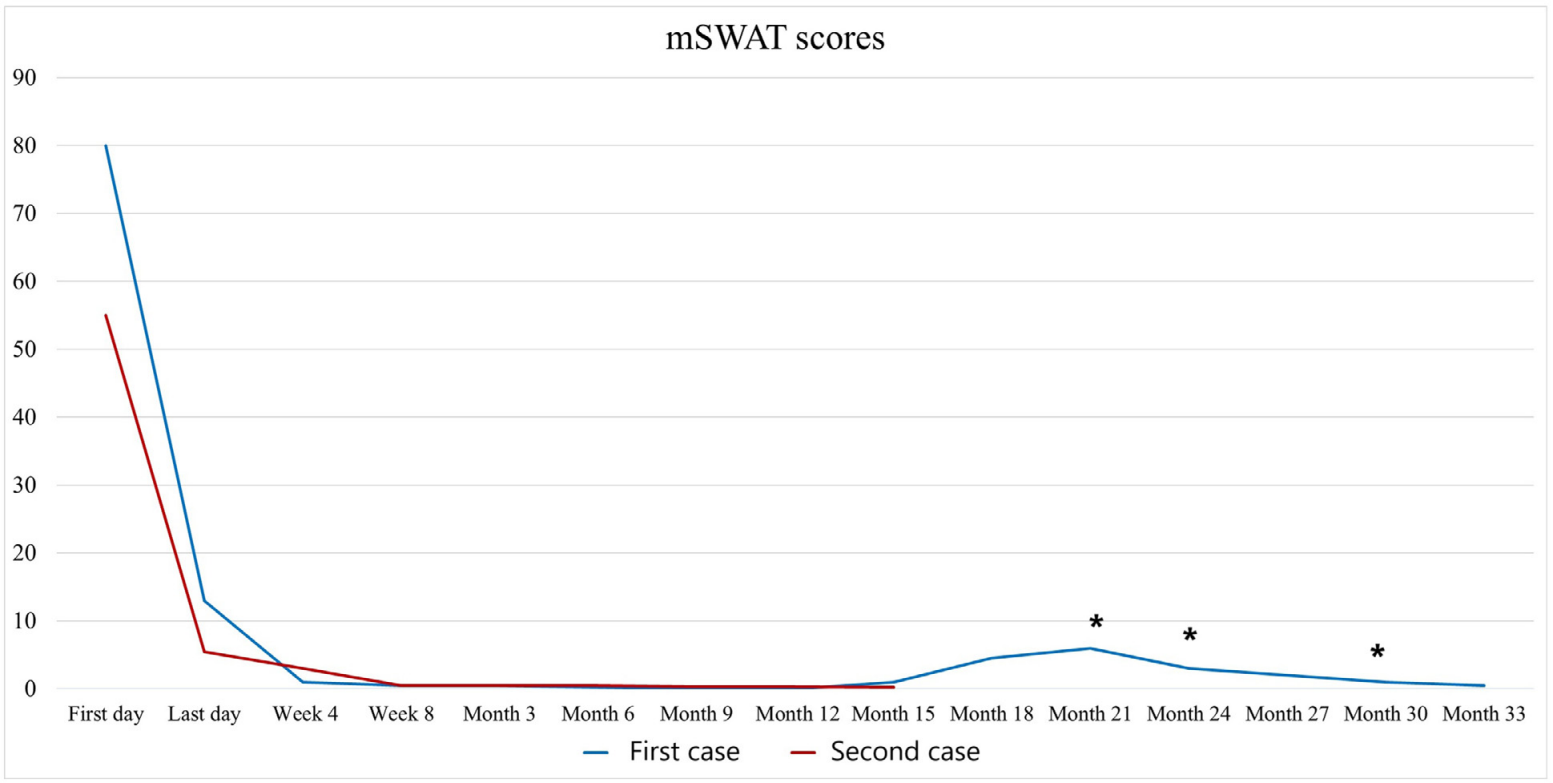
Reduction of cutaneous manifestations with a near-complete response within 4 weeks after total skin electron beam therapy. *Abbreviation:* mSWAT = modified severity weighted assessment tool, * Local radiotherapy.

Two cases with advanced-stage MF reached near complete remission (CR) after low-dose TSEBT plus concurrent and maintenance pegylated IFN alpha-2a (Fig. 1). We obtained informed consent from both patients before RT and IFN administration.

### First case

**Figure 2:**
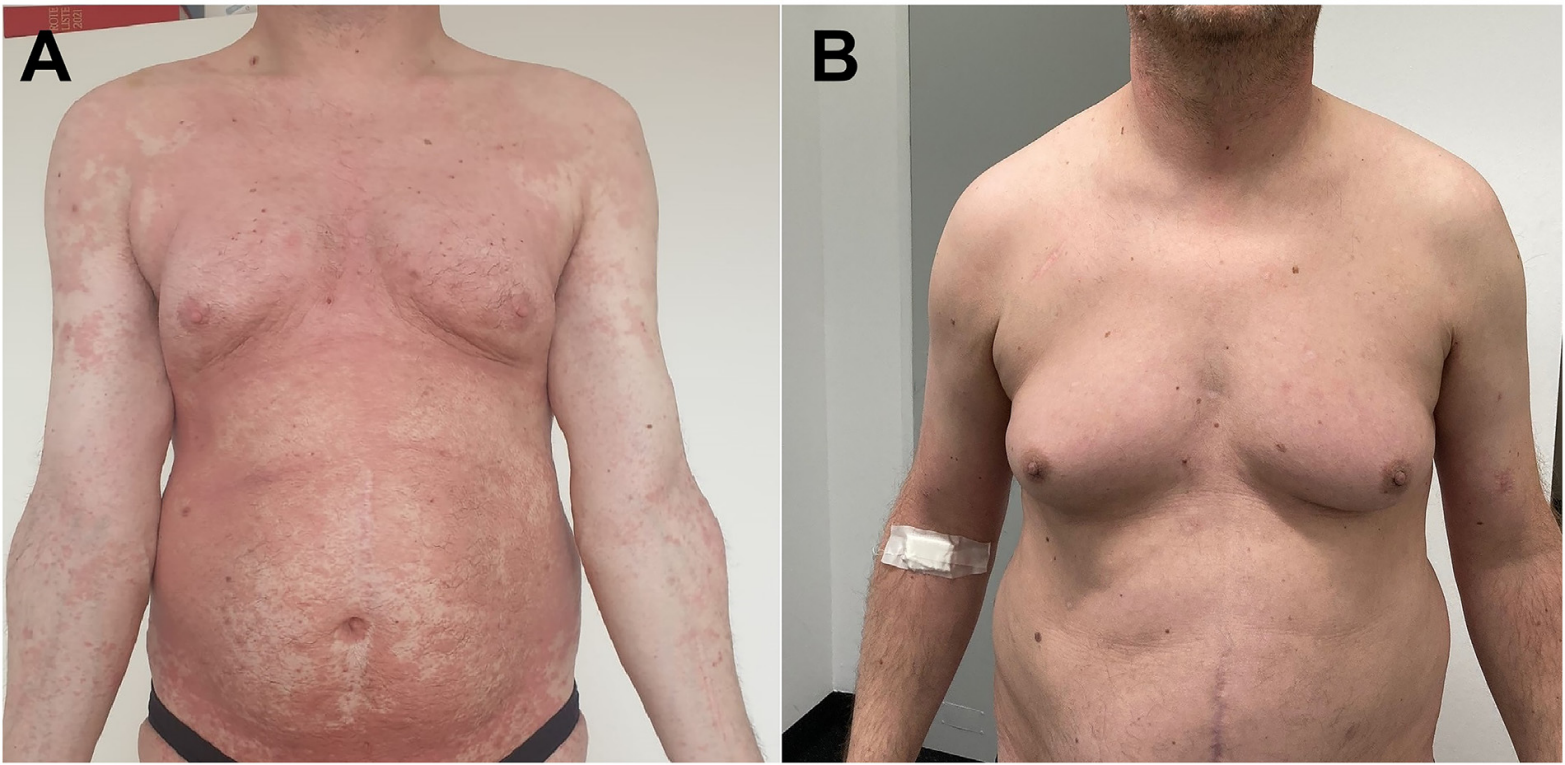
Patient 1 (A) at baseline with a modified severity weighted assessment tool score of 80 and (B) at month 6 with a modified severity weighted assessment tool score of 0.2.

Stage IIIA (T4N1M0B0) 52-year-old male patient with diffuse erythroderma. He had undergone 3 prior therapies (UV-A/UV-B, local RT, and anti-CC chemokine receptor 4 monoclonal antibody mogamulizumab) before receiving low-dose TSEBT and pegylated IFN alpha-2a. This patient received 12 Gy TSEBT with a 1.5 Gy daily fraction (4 fractions per week) and boosts to the soles (8 Gy in 4 fractions) and perineum (12 Gy in 6 fractions) concurrently to TSEBT. The pathologically involved axillary nodes were additionally irradiated with 30.6 Gy in 17 fractions. The weekly IFN alpha-2a with 135 μg was administered sequentially 2 weeks after RT completion. He experienced mild transient erythema and temporary hair loss. Disease assessment showed near-CR after 4 weeks (Fig. 2). Due to discrete small cutaneous relapses, the IFN dose was increased to 180 μg then 270 μg per week combined with multiple ultra–low-dose local RT courses (4 Gy in 2 fractions) to symptomatic lesions, which maintained the clinical response with asymptomatic grade 1 to 2 leucopenia. He remains in response after 33 months of follow up.

### Second case

**Figure 3:**
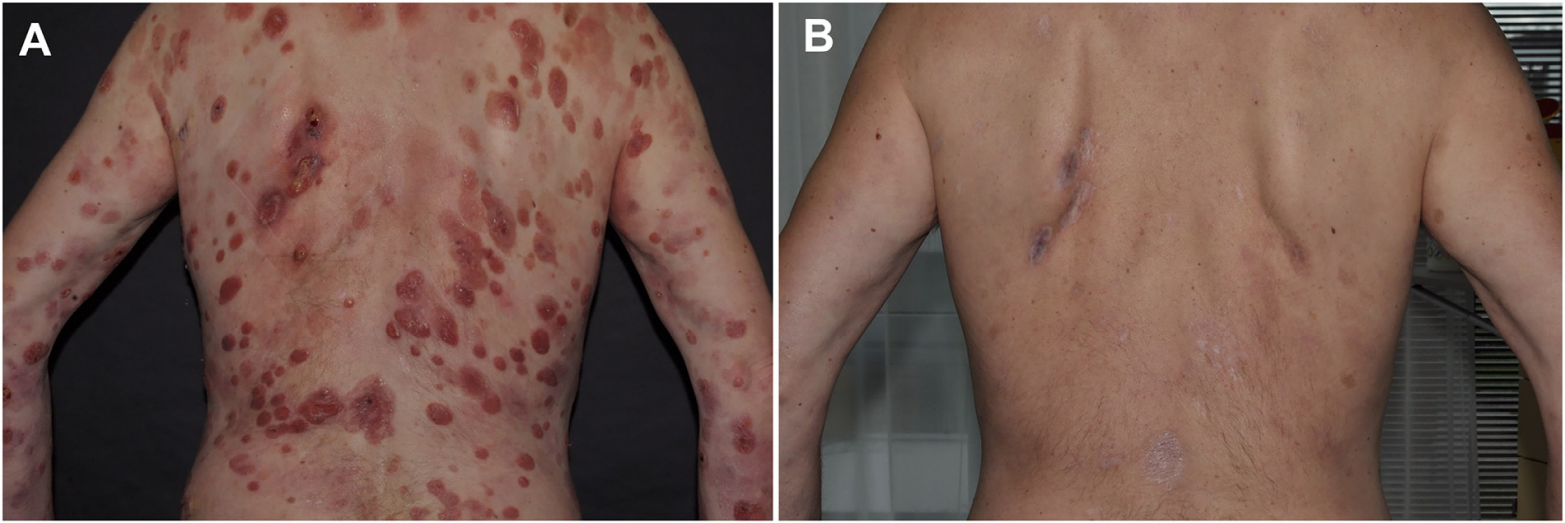
Patient 2 (A) at baseline with a modified severity weighted assessment tool score of 55 and (B) at month 6 with a modified severity weighted assessment tool score of 0.5.

Stage IIB (T3N1M0B0) 57-year-old male patient with diffuse ulcerated tumors and patches. The ulcers were colonized by *Staphylococcus aureus*. The patient received during hospitalization in the University of Dermatology in Minden a triple antibiotic schema (cefuroxim 1.5 g intravenous, metronidazole 0.5 g, and amoxicillin/clavulanic acid 500/125 mg oral 3 times daily for 10 days). The patient had received no systemic therapies before TSEBT and pegylated IFN alpha-2a administration. He began pegylated IFN alpha-2a with 135 μg weekly 2 weeks before RT initiation and received 8 Gy TSEBT with 4 Gy fractions per week simultaneous to the weekly pegylated IFN alpha-2a therapy. No additional local RT was applied during TSEBT because of his rapid therapeutic response (Fig. 3). A rapid near-CR was noted on the last day of radiation. He experienced mild transient erythema, temporary hair loss, and fatigue. He has remained in near-CR after 15 months of follow up. Weekly IFN has been well tolerated with asymptomatic grade 1 to 2 leucopenia.

## Discussion

IFN alpha was first reported as an effective therapy for advanced and heavily pretreated MF nearly 40 years ago. Combinations of IFN alpha with either psoralen plus ultraviolet-A radiation (PUVA) or retinoids to treat MF were evaluated in a German prospective randomized trial.^14^ These combinations displayed high response rates of about 80% with PUV-A and 60% with acitretin. However, the time to response was relatively long (5 months in both arms).^14^ The rate of grade 3 toxicity was lower in the IFN alpha/PUV-A group (12%) compared with the IFN alpha/acitretin group (45%). The most common toxicities are influenza-like symptoms, skin dryness, neurologic/psychiatric disorders, gastrointestinal disorders, and leukopenia. On the other hand, Wagner et al^15^ and Roberge et al^16^ could not detect any survival benefit in patients with MF who were treated with 30 to 36 Gy TSEBT ± IFN. However, nearly all patients suffered from grade 2 or 3 treatment-related dermatitis because of the receipt of relatively high-dose RT and IFN. From a radiobiological perspective, it is well-known that RT doses > 12 Gy upregulate 3 prime repair exonuclease 1, a DNA nuclease that removes cytoplasmic DNA, consequently canceling the RT-induced antitumor immune response.^17^, ^18^, ^19^ Therefore, lower radiation doses might be more beneficial in this immunogenic disease.

In a retrospective study of 12 patients who were administered pegylated IFN alpha-2a at a weekly dose of 135 μg, therapy was safe and required no toxicity-related treatment discontinuations.^20^ In a multicentric retrospective study of 105 patients, pegylated IFN alpha-2a demonstrated 38% partial response (PR) in stage III disease (n = 8). In stage IIB (n = 30), the overall response rate (ORR) was relatively higher and reached 50%. The median duration of clinical benefit (DOCB) was 8 months in both stages. Two-thirds of cases received combined therapy (only 4 patients received TSEBT) with superiority over pegylated IFN alpha-2a alone.^21^ In advanced disease stages, adding low-dose TSEBT to IFN may potentiate the antineoplastic immune reaction, which is essential to avoid relapse or progression.^1^^,^^22^^,^^23^ It is well-known that low-dose TSEBT rapidly reduces patient symptoms and enhances health-related quality of life.^6^^,^^24^ IFN administration before or during RT also augments remission rates via IFN-mediated radiosensitization, which results from an arrested G2 and M stage of the cell cycle.^25^

One of the known risk factors for MF disease progression is superinfection with *Staphylococcus aureus*. Responses to *Staphylococcus aureus* can enhance the neoplastic expansion of tumor cells in MF. Lindahl et al^26^ observed that antibiotic therapy was associated with declined interleukin 2 high-affinity receptors (CD25). In addition, as cutaneous lymphoma advances and neoplastic T cells gather within the cutaneous layers, the tumor microenvironment (TME) converts to type 2 helper T cells (Th2) predominant, with high levels of proinflammatory cytokines that indirectly sustain their growth.^27^ Th2 dominance leads to decreased antineoplastic cells (such as CD8+ T, natural killer, and dendritic cells) and IFN (alpha and gamma).^27^ Exogenous IFN beta administration into tumor tissue seems adequate to boost antigen-specific T cells, leading to CD8+ T cell-dependent regression of neoplastic cells and disease control.^28^

Additional RT can activate type I IFN production and provoke innate and adaptive antitumor immune responses. Locally increased IFN signaling within the TME reverses the suppressive mechanisms and induces antitumor immunity/tumor regression,^28^ while additional IFN therapy increases the cytolytic activity of T cells in vivo and increases cytotoxic T-lymphocyte activity.^28^ Moreover, RT can upregulate the expressions of danger signals, activating ligands for immune cells, neoantigens, and proinflammatory cytokines such as IFN gamma in the TME.^29^, ^30^, ^31^ RT might also augment the cellular various cytokines production encoded by “early response” genes (eg, interleukin 1, 6, 8, and tumor necrosis factor alfa)^32^^,^^33^ and stimulate the proliferation of activated CD8+ T cells.^34^ Afterward, activated CD8+ T cells release IFN gamma, inciting local inflammation.^35^ On the other hand, RT-driven abscopal responses depend on the secretion of type I IFN and rely on CD8+ T cells.^36^ RT upregulates cyclic GMP-AMP synthase-dependent type I-mediated IFN production following mitochondrial DNA accumulation. Also, RT can trigger DNA-driven pathways in host and neoplastic cells, leading to type I IFN production.^37^ In a radiobiological study, tumor diameter was inversely proportional to IFN gamma-producing T cells.^38^ Furthermore, other researchers found that cytoplasmic double-stranded DNA raised more than 10-fold in radiated compared with untreated cells, leading to IFN beta release and upregulation of IFN alpha/beta receptor 1.^17^ The field of translational oncology of MF is emerging, which might provide a better understanding of future diagnostic and therapeutic modalities. More radiobiological studies are warranted to investigate the efficacy of different IFN and radiation doses on the growth of neoplastic cells.

In a previous prospective TSEBT trial with 12 Gy (in 12 fractions),^3^ 33 patients were analyzed, with 21% of patients having stage IIB (n = 7) and 6% having stage IIIA disease (n = 2) without maintenance treatment. The response rate was 96% and 50%, respectively. The overall DOCB was nearly 18 months. Similarly, the UK cutaneous lymphoma group^39^ reports a response rate of 97% in stage IIB (n = 33) and 50% in stage III disease (n = 12) following 12 Gy in 8 fractions. At the same time, the DOCB was only 9 months and 11 months, respectively. Although, 14 of 103 patients received maintenance treatment (5 received IFN alpha). In the prospective TSEBT trial with 10 Gy (in 10 fractions),^5^ 21 patients were analyzed, with 24% of patients having stage IIB (n = 5) and 5% having stage IIIA disease (n = 1). Only 1 patient received non-pegylated IFN as maintenance treatment. The response rate was 100%, respectively. The overall DOCB was nearly 6 months (range, 3-13.5). Recently, TSEBT with 8 Gy (in 2 fractions) proved safe and effective with only 2 hospital visits. The median DOCB with an ultrahypofractionated regimen was 12 months.^2^ Ultra–low-dose TSEBT with 4 Gy (in 4 fractions) induces a response in 3 patients with stage IIB disease. However, the duration of response ranged between 1 and 3 months.^40^

To our knowledge, this is the first well-documented report on the combination of low-dose TSEBT with weekly pegylated IFN alpha-2a. This combined modality seems safe, improves the clinical response, and is associated with good patient compliance and quality of life.^9^, ^21^, ^41^, ^42^ We did not observe grade 3 or 4 toxicities compared with the historical data on the IFN and RT combination, with few hospital visits. However, our patients received different radiation and IFN doses. A prospective data collection of real-world practice is urgently needed to identify the clinical and treatment parameters and lower radiation doses (such as ultra–low-doses with 2 or 4 Gy) influencing the outcomes of patients with MF. Our prospective observational data collection to evaluate the role of TSEBT-IFN in MF is underway (German Clinical Trials Register: DRKS00031975).

## Conclusions

Low-dose TSEBT plus maintenance IFN alpha-2a seems safe and has reasonable activity in 2 cases of advanced-stage MF. An observational study to explore the role of TSEBT combined with IFN alpha-2a in MF is underway.

## Disclosures

None.
